# Quality assurance and its impact on ovarian visualization rates in the multicenter United Kingdom Collaborative Trial of Ovarian Cancer Screening (UKCTOCS)

**DOI:** 10.1002/uog.14929

**Published:** 2016-02-02

**Authors:** A. Sharma, M. Burnell, A. Gentry‐Maharaj, S. Campbell, N. N. Amso, M. W. Seif, G. Fletcher, C. Brunell, G. Turner, R. Rangar, A. Ryan, I. Jacobs, U. Menon

**Affiliations:** ^1^ Gynaecological Cancer Research Center, Department of Women's Cancer, Institute for Women's HealthUniversity College LondonLondonUK; ^2^ Department of Gynaecological OncologyUniversity Hospital of WalesCardiffUK; ^3^ Create Health ClinicLondonUK; ^4^ Institute for Translation, Innovation, Methodology and Engagement, School of MedicineCardiff UniversityCardiffUK; ^5^ Academic Unit of Obstetrics and GynaecologySt Mary's HospitalManchesterUK; ^6^ Department of RadiologyUniversity College London HospitalsLondonUK; ^7^ Department of RadiologyRoyal Derby HospitalDerbyUK; ^8^ Northern Gynaecological Oncology CenterQueen Elizabeth HospitalGatesheadUK; ^9^ University of New South WalesSydneyNSWAustralia

**Keywords:** ovarian cancer screening, ovarian visualization, postmenopausal women, quality assurance, transvaginal scan, UKCTOCS

## Abstract

**Objective:**

To describe the quality assurance (QA) processes and their impact on visualization of postmenopausal ovaries in the ultrasound arm of a multicenter screening trial for ovarian cancer.

**Methods:**

In the United Kingdom Collaborative Trial of Ovarian Cancer Screening, 50 639 women aged 50–74 years were randomized to the ultrasound arm and underwent annual transvaginal ultrasound (TVS) examinations. QA processes were developed during the course of the trial and included regular monitoring of the visualization rate (VR) of the right ovary. Non‐subjective factors identified previously as impacting on VR of the right ovary were included in a generalized estimating equation model for binary outcomes to enable comparison of observed vs adjusted VR between individual sonographers who had undertaken > 1000 scans during the trial and comparison between centers. Observed and adjusted VRs of sonographers and centers were ranked according to the highest VR. Analysis of annual VRs of sonographers and those of the included centers was undertaken.

**Results:**

Between June 2001 and December 2010, 48 230 of 50 639 women attended one of 13 centers for a total of 270 035 annual TVS scans. One or both ovaries were seen in 228 145 (84.5%) TVS scans. The right ovary was seen on 196 426 (72.7%) of the scans. For the 78 sonographers included in the model, the median difference between observed and adjusted VR was −0.7% (range, −7.9 to 5.9%) and the median change in VR rank after adjustment was 3 (range, 0–18). For the 13 centers, the median difference between observed and adjusted VR was −0.5% (range, −2.2 to 1%), with no change in ranking after adjustment. The median adjusted VR was 73% (interquartile range (IQR), 65–82%) for sonographers and 74.7% (IQR, 67.1–79.0%) for centers. Despite the increasing age of the women being scanned, there was a steady decrease in the number of sonographers with VR < 60% (21.4% in 2002 vs 2.0% in 2010) and an increase in sonographers with VR > 80% (14.3% in 2002 vs 40.8% in 2010). The median VR of the centers increased from 65.5% (range, 55.7–81.0%) in 2001 to 80.3% (range, 74.5–90.9%) in 2010.

**Conclusions:**

A robust QA program can improve visualization of postmenopausal ovaries and is an essential component of ultrasound‐based ovarian cancer screening trials. While VR should be adjusted for non‐subjective factors that impact on ovarian visualization, subjective factors are likely to be the largest contributors to differences in VR. © 2015 The Authors. *Ultrasound in Obstetrics & Gynecology* published by John Wiley & Sons Ltd. on behalf of the International Society of Ultrasound in Obstetrics and Gynecology.

## INTRODUCTION

The safe and effective delivery of a cancer‐screening program depends on the provision of a high‐quality service, continued development of the personnel delivering and running the service and a systematic approach linking all the activities involved in the identification of the cancer^1^. To monitor and evaluate these processes, quality assurance (QA) programs are essential. They are major contributors to the success of the breast and cervical screening programs in the UK. QA processes are often developed during the course of screening trials to facilitate future clinical implementation if appropriate. This is especially relevant to screening tests with a significant subjective element such as transvaginal ultrasound (TVS), which is integral to screening strategies for ovarian cancer^2^.

In the last decade, four major ovarian cancer screening trials with different screening protocols have evaluated the efficacy of TVS as a screening test, with variable results^3^, ^4^, ^5^, ^6^. The single‐center Kentucky Ovarian Cancer Ultrasound Screening study reported a possible survival benefit^3^, whereas screening using a combination of TVS and CA 125 did not result in a stage shift in the Japanese Shizuoka Cohort study^4^ or demonstrate a mortality benefit in the USA Prostate, Lung, Colorectal and Ovarian (PLCO) screening trial^5^. The mortality impact of screening in the United Kingdom Collaborative Trial of Ovarian Cancer Screening (UKCTOCS)^6^, which completed screening in 2011, is awaited.

One of the concerns regarding the use of ultrasound in screening has been the considerable interobserver variability reported in ovarian visualization in large screening studies, and the fact that the accuracy of interpretation depends on the operator's experience^2^. Here we describe the methods used to ensure delivery of high‐quality ultrasound scanning in a multicenter setting, to define parameters for QA during TVS examination of postmenopausal ovaries and to assess the impact of QA processes over time on visualization rates (VR) of the ovary for individual sonographers and regional centers, after adjustment for non‐subjective factors that can impact on VR^2^.

## METHODS

### Participants, design and follow‐up within the UKCTOCS

Details regarding volunteers and design of the trial have been described previously in greater detail^6^, ^7^. In brief, of the 202 638 women recruited to the trial, 50 639 were randomized to the ultrasound arm and underwent annual TVS screening between October 2001 and December 2011. Transabdominal ultrasound (TAS) was undertaken only when TVS was declined by the participant. The examinations were performed by sonographers at 13 participating regional centers. All scan data were entered contemporaneously on a central web‐based trial management system. Women were followed up for a diagnosis of cancer through a ‘flagging study’ via the Health and Social Care Information Center (formerly the UK Office of National Statistics) and through postal questionnaire sent 3½ years after the women were randomized to the trial. This analysis was restricted to all women in the ultrasound group who had a scan by 31 December 2010. The UKCTOCS study was approved by the UK North West Multicentre Research Ethics Committees (North West MREC 00/8/34). It is registered as an International Standard Randomized Controlled Trial, number ISRCTN22488978.

### Ultrasound protocol

The ultrasound protocol addressed all aspects of scanning during the trial. It included the standard operating procedure (SOP) on scanning the pelvis in postmenopausal women, which provided detailed guidance on the correct techniques to accurately examine the pelvis (starting with the uterus in the sagittal and transverse sections, reducing the depth and following the ovarian ligament out to the pelvic sidewall) and to identify the ovary based on its ultrasound appearance (small static hypoechoic area between the iliac vessel and ovarian ligament, adjacent to the iliac vessel anywhere along its length, and usually in close proximity to the uterus). Measurement of three ovarian diameters in two planes for the automated calculation of ovarian volume by the trial management system was detailed, as were suggestions for image optimization (abdominal palpation to displace the bowel, using the dual screen, manipulating the image to view the ovary in the middle of the screen, increasing the depth or using zoom to magnify the image of the ovary, positioning focus correctly, using frequency to improve resolution at required depth, slow frame rates and using the scroll facility to obtain the best frame after freezing). The classification and reporting of ovarian/adnexal lesions were standardized. Algorithms for the management of the findings are detailed elsewhere^6^.

If the sonographer, the trial center team or the center's lead consultant had concerns regarding an ultrasound examination, they could request a review of the images by a senior UKCTOCS investigator with ultrasound expertise at the coordinating center.

### Ultrasound machines

Dedicated centrally procured ultrasound machines with service contracts were used across the regional centers. At all centers, the Kretz SA9900 ultrasound machine (Medison, Seoul, South Korea) was used between 2001 and 2007, followed by the Medison Accuvix ultrasound machine from 2007 to 2011. At the beginning of the trial, and when ultrasound machines were upgraded, centers were visited to ensure uniform settings were used on all machines. The ultrasound machines were calibrated and maintained regularly under a centrally agreed UKCTOCS service contract.

### Key components of the QA process

#### *Ultrasound subcommittee*

Implementation of the ultrasound arm and set up and running of the QA processes were overseen initially by the team at the coordinating center and the trial investigators with ultrasound expertise. In 2006, a formal ultrasound subcommittee was formed. Chaired by the trial coordinator, the committee included senior trial gynecologists with expertise in gynecological imaging, radiologists experienced in abdominal and pelvic imaging, a national lead sonographer (NLS) for the trial, senior sonographers from regional centers and the coordinating center team, who were involved in day‐to‐day monitoring of the ultrasound protocol and QA process. The subcommittee managed the training and set up an accreditation process for sonographers, monitoring of adherence to the ultrasound protocol, fail‐safe monitoring and further development of the QA processes. They also had oversight of the logistics involved in implementation of the ultrasound arm of the trial. The subcommittee met three times a year with e‐mail updates from the NLS on a monthly basis.

#### *National lead sonographer (NLS)*

The NLS was a senior sonographer (superintendent level) with extensive experience in gynecological scanning and management of sonographers. She was appointed to the coordinating center team to oversee the day‐to‐day delivery of ultrasound across regional centers and take on QA management and execution of the ultrasound subcommittee's recommendations. Central to this were assessing and addressing the training needs of each sonographer, developing a system for their accreditation and re‐accreditation, running weekly fail‐safe checks on scan reports to monitor discrepancies or errors and implementing QA monitoring. In addition, the NLS worked with the regional centers to manage the logistics of delivering an average of 3000 scans per center per year following the end of recruitment. In her absence, the clinical research fellow ran the fail‐safe checks and QA monitoring.

#### *Personnel scanning in trial*

Type 1 sonographers (certified sonographers, trained midwives or doctors in the National Health Service (NHS) trained in gynecological scanning) performed TVS as a first line‐test (level 1) in the ultrasound arm^6^. If the results of the level 1 scan were normal, the women were returned to annual screening and scanned the following year by a type 1 sonographer. However, on detection of an abnormality on the level 1 scan, a repeat scan (level 2) was arranged^6^. These were performed by type 2 sonographers (senior sonographers, usually at superintendent level, experienced gynecologists or radiologists) with expertise in gynecological scanning^6^.

#### *Induction of sonographers*

Each sonographer commencing scanning in the trial was required to submit their curriculum vitae giving details of their qualifications and scanning experience. They received a current copy of the scanning protocol. On‐site training was provided to familiarize each with the protocol and recommended methods of scanning before scanning independently on the trial. The NLS, designated senior trial gynecologists or radiologists with ultrasound expertise or, occasionally, a delegated experienced local sonographer, supervised the new sonographer for a minimum of two scanning sessions, assessed competency and then authorized the sonographer to scan independently in the trial. A practical assessment was arranged 3 months after the sonographers joined the trial and this was later incorporated into the accreditation process.

#### *Ongoing training of sonographers*

Central training days, often spanning weekends, were organized twice a year for sonographers, nurses and other members of the regional center teams. This training included talks on recommended and standardized scanning techniques, definitions of key terms, quality checks and the ultrasound protocol. A mandatory component was a 2‐h case‐based discussion session that included both screen‐detected and screen‐missed (interval) cancers with an emphasis on the availability of a second opinion when faced with equivocal scan findings. It was obligatory for all sonographers to attend at least one study day per year. In addition, all centers were visited at the start, and on a regular basis during the course of the trial, by senior investigators with ultrasound expertise, accompanied by the clinical fellow or NLS. These visits included formal practical training sessions, talks to reinforce key messages and one‐to‐one scanning and assessment, especially of those identified on QA monitoring to have ovarian VRs below 60%.

The coordinating center also circulated newsletters to the sonographers and regional center nurses, communicating updates and news from the regional centers.

Accreditation for individual sonographers was initiated in 2008 and comprised completion of a questionnaire to demonstrate understanding and knowledge of the UKCTOCS protocol, assessment of VR by review of scan data over a 3‐month period, practical assessment by the NLS, which included scanning of a minimum of five volunteers, and submission of nine sets of images for central review by the NLS and a subcommittee member with expertise in pelvic scanning.

#### *Ultrasound data collection and data entry*

Scan data regarding visualization of the ovaries, reasons for non‐visualization of the ovaries, morphological findings of the ovary, details of any abnormality seen, endometrial thickness measurements, presence of free fluid in the pouch of Douglas and any other abnormalities were recorded on a standard data form. In addition, the overall pictorial impression of abnormal morphology was collected initially using the Kentucky pictorial representation of morphology index and subsequently, from 2004, using the International Ovarian Tumor Analysis Group pictorial classification^8^, ^9^.

The sonographer or a member of the local team collated and entered the information online contemporaneously on a dedicated ultrasound reporting section of the web‐based trial management system^7^. The coordinating center provided on‐site training and written instructions on how to enter the data and conducted regular on‐site visits to monitor and audit data entry on the trial management system to ensure accuracy of entries.

Following entry of scan findings on the trial management system, there was central automated classification of scan results based on visualization, morphology and size of simple cysts and recommended management^6^. Results letters were sent to the volunteers directly from the coordinating center, as were appointment letters for their next scan. There was daily regional monitoring of the automated management decisions and weekly central monitoring of any data discrepancies, with editing of incorrect entries and update of management decisions if required. All errors in data entry were fed back immediately and discussed with the regional center team coordinators and individual sonographers.

#### *Fail‐safe monitoring*

Several monitoring mechanisms were implemented. These were overseen by the clinical fellow or the NLS, who ran a series of data queries and read all free text entered on the ultrasound report to detect: (1) discrepancies between the notes and the mandatory classification fields; (2) missing ovarian dimensions when ovaries were visualized; (3) missing descriptions of complex masses; (4) apparent errors in measurement; and (5) incidental findings. All queries were investigated by contacting the regional center team, who reviewed the written scan report and the images as appropriate. The NLS or fellow then corrected data entries on the trial management system. The regional center nurses were also alerted to ensure that all women with significant incidental findings had been referred appropriately to their general practitioner or the hospital team as per locally agreed guidelines.

#### *Archiving and review of images*

Gray‐scale images of ultrasound scans performed during the trial were archived. The protocol defined the views to be stored. For a normal scan, transverse and sagittal views of each normal ovary with measurements and a section through the uterus, including the endometrial thickness measurement, were stored. For an abnormal scan, images with measurements of any mass in two perpendicular planes were stored. If Doppler was used, the frames demonstrating the waveforms and calculations and the images of any incidental abnormalities were stored. Images were archived initially on the ultrasound machines at the centers and were then transferred weekly or biweekly (depending on volumes) on a magneto‐optical disc to the coordinating center for central archiving. Regular reviews of images of interval cancers were conducted, with feedback given to individual sonographers and discussion at the annual ultrasound meetings. In addition, the NLS or senior members of the ultrasound subcommittee reviewed all images for which concern was raised on fail‐safe monitoring of the scan reports.

### Quality assurance monitoring

Since visualization of the ovary is essential to identify any morphological abnormality of the ovary, it was considered the most important metric for QA in the trial. The sonographers were required to indicate whether each ovary was seen; was not seen but a good view of the pelvis was obtained; was not seen with a poor view of the pelvis; or was not seen owing to previous oophorectomy. It was decided that ‘visualization of the ovary’ would be the primary QA measure, as this could be verified by review of archived images when required. As we did not observe a substantial difference in VR between the right and left ovary on TVS, monitoring was based on the VR of the right ovary^10^. Unadjusted VRs of the right ovary were calculated on a 6‐month basis for individual sonographers undertaking more than 1000 scans during the trial period and for individual regional centers. We set a standard for observed unadjusted VR of the right ovary of 60%, based on data derived from the first 2 years of scanning in UKCTOCS and the report of VRs of one or both ovaries (data for individual ovaries were not available) from the Kentucky screening study^11^. Other metrics used for QA monitoring included median volume of normal ovary and missing or incorrect data in key fields, such as ovarian measurements. Reports incorporating coded individual and center data were circulated to all involved in the trial. Center leads were informed of their regional codes so that they could discuss the report in detail with their team of sonographers. In addition, the NLS contacted individual outliers for targeted training and worked with them to improve their VR.

### Statistical analysis

Descriptive statistics were used to describe baseline characteristics of the women according to which sonographer performed the scan and to the center at which they were examined.

The VR of the right ovary was adjusted for the strongly significant (*P* < 0.01) non‐subjective factors identified in a previous study (age, hysterectomy, oophorectomy with intact uterus, age at menopause, tubal ligation, body mass index)^10^. For individual women, the ability to visualize the ovary is likely to be correlated across annual scans. The VR was therefore modeled, taking into account clustering of outcomes. To provide population‐averaged rather than individual‐level effects of covariates on visualization, we fitted a generalized estimating equation model for binary outcomes, with a logit link function and an ‘exchangeable’ correlation structure. Such a model is similar to a standard logistic regression model but with a specified intraperson correlation structure. Sonographers who had performed more than 1000 scans in total between 2001 and 2010 were included as separate fixed effects in the model. These sonographers were compared with a reference group consisting of those who had performed ≤ 1000 scans. A separate model that included the individual center effects (but with no sonographers) was also fitted. Age at first scan indicated the actual age effect, whereas scan year reflected the trend in ultrasound performance. Odds ratios for individual sonographers and centers reflected performance relative to the reference group. Comparison of model‐based adjusted VR with observed VR for individual sonographers and centers helped to indicate how the differences in the non‐subjective factors impacted on the VR of sonographers and centers. It was an indicator of the need for adjustment of VR in this setting. Rankings for the observed and adjusted VRs (1 = highest VR) reflected the impact of these adjustments.

Annual trends of observed VR of the right ovary between 2001 and 2010 were plotted for individual sonographers and regional centers. The VRs calculated for each center included all scans performed at the center during that calendar year. Annual observed VR of the right ovary for individual sonographers was limited to sonographers who had undertaken > 1000 level‐1 TVS examinations overall and > 100 during that calendar year.

## RESULTS

Between 11 June 2001 (date of commencement of scanning in the trial) and 31 December 2010, across 13 regional centers, 48 230 of the 50 639 women in the ultrasound screening arm attended at least one screening examination and underwent a total of 293 732 annual scans. Of these, 23 697 were TAS examinations and were excluded from the analysis. The remaining 270 035 scans comprised 267 036 TVS examinations and 2999 in which both TVS and TAS were performed. The right ovary was seen in 196 426 (72.7%) scans, and was not visualized but there was a good view of the pelvis in 62 683 (23.2%) and a poor view of the pelvis in 6429 (2.4%); a previous unilateral oophorectomy was noted in 4493 (1.7%). Four scans were unrecorded. The VR of the left ovary was 69.7% (188 347/270 035) and the VR of one or both ovaries was 84.5% (228 145/270 035). The 270 035 annual scans between 2001 and 2010 included in the trial were performed by 294 sonographers. However, 222 666 (82.5%) were undertaken by 78 sonographers who had performed > 1000 scans at a center. The remaining scans (47 369) formed the reference group in the model comparing individual sonographers.

We reported previously that age at scan, age at menopause, being overweight, previous hysterectomy, previous sterilization and unilateral oophorectomy with intact uterus were significant predictors of VR^10^. These were used in the longitudinal analysis along with the scan year to reflect the overall trend in scanning performance (Table S1). The mean baseline characteristics of the women scanned by the 78 individual sonographers with > 1000 scans showed more variability than was found in the mean baseline characteristics of the women scanned in the different centers but with no evidence of any large systematic differences. For example, the mean age ranged between 59.5 and 63.3 years and hysterectomy rate ranged between 14.2% and 22.0% (Table S2).

The median adjusted VR for sonographers was 73% (interquartile range (IQR), 65–82%). The observed VR was outside the 95% CI of the adjusted VR for 38/78 (48.7%) sonographers included in the model. However the median difference between observed and adjusted VRs was only −0.7% (range, −7.9 to 5.9%). Adjustment of VR changed the attainment of the trial standard VR of 60% in only 4/78 (5.1%) sonographers, increasing the VR to ≥ 60% in two and decreasing the VR to < 60% in two. However, the impact of adjustment on VR ranking was greater, with a median change in rank of 3 (range, 0–18).

The baseline characteristics of women scanned at the 13 centers (Table S3) were similar, except for Center G, which had the lowest hysterectomy rate (13.6% *vs* overall average of 18.1%; range, 13.6–22.0%), the lowest proportion of overweight or obese women, the second lowest rate of tubal ligation and the highest rate of infertility treatment. The median adjusted VR for the 13 centers was 74.7% (IQR, 67.1–79%), all centers having an adjusted VR above the minimum standard of 60%. The median difference between observed and adjusted VR for the 13 regional centers was −0.5% (range, −2.2 to 1%), with no change in ranking for any center (Table S4).

**Figure 1:**
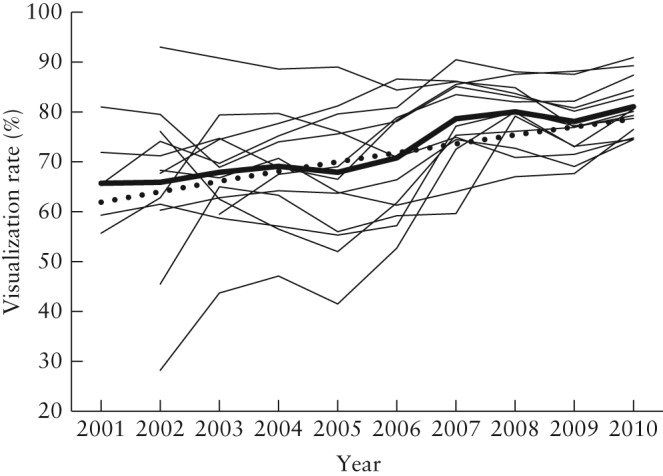
Annual visualization rates (VR) of the right ovary by transvaginal ultrasound in 48 230 postmenopausal women attending one of 13 regional centers from 2001–2010. Thick lines indicate adjusted VR (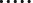) and median observed VR (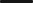). Thin lines represent each of the 13 centers.

Given the small differences between the observed and adjusted VRs in our trial, the former was used to determine trends over time. The annual observed VR of individual sonographers who had performed more than 1000 trial scans and of the 13 centers are detailed in Table 1 and Figure 1, respectively. The number of sonographers with VR < 60% (21.4% in 2002 *vs* 2.0% in 2010) decreased and those with VR > 80% (14.3% in 2002 *vs* 40.8% in 2010) increased over time, a trend that was more pronounced after 2006. This is reflected in the steady increase in the annual observed VR of ovaries at individual centers (Figure 1). The median center‐observed VR increased from 65.5% (range, 55.7–81.0%) in 2001 to 80.3% (range, 74.5–90.9%) in 2010. The median center‐observed VR in 2003, after all 13 participating centers became active, was 66.8%. In 2008 all centers had a VR of > 60%, seven of the 13 centers achieving overall VRs of > 80%.

**Table 1:** Annual observed ovarian visualization rates (VR) of right ovary for 78 sonographers who had each performed > 1000 scans during United Kingdom Collaborative Trial of Ovarian Cancer Screening

	Year									
Parameter	2001	2002	2003	2004	2005	2006	2007	2008	2009	2010
Number of sonographers	1	14	33	54	57	64	65	64	54	49
Observed VR										
< 50%	—	3 (21.4)	6 (18.2)	9 (16.7)	8 (14.0)	9 (14.1)	5 (7.7)	1 (1.6)	—	—
50–60%	—	—	—	5 (9.3)	10 (17.5)	8 (12.5)	3 (4.6)	5 (7.8)	4 (7.4)	1 (2.0)
60–80%	1 (100)	9 (64.3)	15 (45.5)	20 (37.0)	20 (35.1)	30 (46.9)	34 (52.3)	35 (54.7)	30 (55.6)	28 (57.1)
> 80%	—	2 (14.3)	12 (36.4)	20 (37.0)	19 (33.3)	17 (26.6)	23 (35.4)	23 (35.9)	20 (37.0)	20 (40.8)

Data are given as *n* or *n* (%).

## DISCUSSION

Our report is the first detailed description of QA processes required for delivering TVS in large multicenter ovarian cancer screening trials or programs and for evaluating their impact on ovarian visualization. Our results show that the QA processes developed, together with regular monitoring, can lead to a high VR of postmenopausal ovaries and ensure continual improvement in VR over time. The VR showed a steady increase over the years, with the majority of sonographers and all centers achieving a VR of individual ovaries of > 60%. The differences in VR partly reflect the differing scanning abilities of individual sonographers. It is important to highlight that ovarian VR is not a perfect metric, as it underestimates pelvic visualization by not defining scans as ‘visualized’ when ovaries are not seen but there is a good view of the pelvic sidewall or when unilateral ovaries are not seen owing to previous oophorectomy.

A key finding was that differences between observed and adjusted VRs were small. Adjustment had the greatest impact on individual sonographer ranking. This probably results from differences in non‐subjective factors being small between centers and being more pronounced between individual sonographers. The persistent difference in adjusted VR confirms that subjective factors, i.e. individual skill, attention to detail and experience, are major contributors, in keeping with previous reports that subjective assessment of the gray‐scale image has the highest accuracy^12^, ^13^, ^14^. It is likely that QA processes coupled with central monitoring and targeted training impacted on both non‐subjective and subjective factors.

Ovarian visualization is key to identifying morphological abnormalities that may be indicative of ovarian cancer. Our VR of one or both ovaries was 84.5% in an analysis of 270 035 TVS examinations. This was higher than that reported in the PLCO trial^5^ which, like UKCTOCS, was a multicenter trial in which postmenopausal women underwent annual screening. Bodelon *et al.* 15 reported an ovarian VR (one or both ovaries visualized) of 53% in their most recent analysis of 102 787 scans involving 29 321 women. Both subjective and non‐subjective factors could have contributed to these differences. The rates probably reflect differences in delivery of ultrasound and may have contributed to differences in the stage (UKCTOCS Stages I/II: 48% *vs* PLCO Stages I/II: 22%) of screen‐detected cancers on prevalence screening in the two trials^5^, ^6^. Our VR was similar to that reported in the Kentucky ovarian cancer screening study (84%), in which 25 327 women underwent a total of 120 569 scans during an 18‐year period^3^. This study, unlike UKCTOCS, was limited to a single center with scanning performed by a small group of experienced personnel. Their cohort included a proportion of younger premenopausal women, in whom the presence of follicles makes ovaries easier to visualize. Neither study reported on the VR of individual ovaries as we have done for QA monitoring in UKCTOCS. Higher ovarian VR (right, 84.1%; left, 82.4%) was reported in a retrospective 5‐year audit of 6649 postmenopausal women who underwent TVS as part of clinical evaluation in a gynecological ultrasound department^16^. The higher rates most probably reflect the scanning expertise available at tertiary centers and also the higher incidence of adnexal pathology (which may make visualization easier) in a clinical, compared to an asymptomatic, cohort attending screening.

In UKCTOCS, sonographers performed the annual level 1 scans. This reflects the practice in the UK NHS, in which sonographers perform the vast majority of pelvic ultrasound scans. It would be difficult to find the resources required for expert gynecologists to deliver primary screening in a national screening program or large multicenter trial involving > 50 000 scans yearly. However operator experience is crucial for accurate interpretation^2^ and patients with adnexal masses undergo fewer operations and a greater number of minimally invasive procedures, and experience shorter hospital stays, when scanned by experienced gynecologists than do those scanned by sonographers^17^. Hence, level 2 scans following the detection of an abnormality were performed by a senior sonographer or expert gynecologist or radiologist.

Individual sonographer and center VRs increased over time despite the increasing age of the participants. This could partly be explained by the increasing experience of the sonographers, some of whom continued to participate in the trial for many years. However, the ongoing QA processes with their focus on sonographer training and support, regular monitoring with feedback and targeted training are likely to have contributed significantly to this increase. The SOP for examining the pelvis in postmenopausal women was introduced to sonographers at induction. It reaffirmed the principles of TVS scanning and emphasized a systematic approach to pelvic examination, identification of ovaries and optimization of images. The NLS as a quality‐control manager was key to the interaction with sonographers. In addition, the introduction of accreditation contributed to improving ultrasound quality. The British Society for Gynaecological Imaging has adopted the UKCTOCS scanning SOP as guidance for good practice and incorporated the accreditation scheme with modifications into the Society's continuing professional development program.

This report demonstrates the benefits of a formal QA process and outlines the procedures to be implemented for multicenter ultrasound screening. By introducing training days and familiarizing sonographers with the scanning protocol and SOPs, uniformity in scanning postmenopausal ovaries and interpreting gray‐scale ultrasound images was maintained. The leadership provided by the subcommittee and the attention to detail and sonographer support and training provided by the NLS were key to the sustained improvement in VR. The process of accreditation was of immediate use to those who wished to maintain standards in pelvic scanning.

Limitations include the fact that QA processes were set up in the course of the trial and the NLS was appointed only midway through the trial. At the start of the trial the standard for VR of the right ovary was set at 60%, but the improvement over time suggests that this could have been set higher. In addition, independent review of the archived images to assess visualization of the ovaries is yet to be completed.

In conclusion, if there are robust centrally implemented and monitored QA processes, ovarian VR as a performance indicator of the quality of ultrasound screening will improve with time, despite adverse factors such as aging. While VR should be adjusted for non‐subjective factors that impact on ovarian visualization, it is likely that subjective factors will be the largest contributors to VR differences.

## DISCLOSURES

U.M. and I.J. have a financial interest through Abcodia Ltd in the third party exploitation of the trial biobank. During part of the trial, I.J. had a consultancy arrangement with Becton Dickinson in the field of tumor markers. The UKCTOCS trial was core funded by the Medical Research Council, Cancer Research UK, and the Department of Health, with additional support from the Eve Appeal, Special Trustees of Bart's and the London, and Special Trustees of University College London Hospital. The researchers at University College London were supported by the National Institute for Health Research University College London Hospitals Biomedical Research Center. The researchers are independent from the funders.

## Supporting information


**Table S1** Generalized estimating equation model for binary outcomes for comparison of observed and adjusted visualization rates (VR) of the right ovary on ultrasound for 78 individual sonographers who had performed >  1000 scans during the United Kingdom Collaborative Trial of Ovarian Cancer Screening, including non‐subjective factors identified previously as impacting on VR

**Table S2** Mean baseline characteristics of 48 230 postmenopausal women scanned by 78 individual sonographers with >  1000 scans performed during the United Kingdom Collaborative Trial of Ovarian Cancer Screening

**Table S3** Mean baseline characteristics of 48 230 postmenopausal women scanned at one of 13 regional centers during the United Kingdom Collaborative Trial of Ovarian Cancer Screening

**Table S4** Generalized estimating equation model for binary outcomes for comparison of observed and adjusted visualization rates (VR) of the right ovary on ultrasound at 13 regional centers participating in United Kingdom Collaborative Trial of Ovarian Cancer Screening, including non‐subjective factors identified previously as impacting on VR
