# Supplementary material for: Quality assurance and its impact on ovarian visualization rates in the multicenter United Kingdom Collaborative Trial of Ovarian Cancer Screening (UKCTOCS)
Source: Ultrasound Obstet Gynecol. 2016 Feb 2;47(2):228–35. doi: 10.1002/uog.14929 (PMC4755159; doi:10.1002/uog.14929)
Supplement: Supplementary file 2 — Table S2 Mean baseline characteristics of 48 230 postmenopausal women scanned by 78 individual sonographers with > 1000 scans performed during the United Kingdom Collaborative Trial of Ovarian Cancer Screening [file UOG-47-228-s002.docx]

| **Table S2** Mean baseline characteristics of 48 230 postmenopausal women scanned by 78 individual sonographers with > 1000 scans performed during the United Kingdom Collaborative Trial of Ovarian Cancer Screening | | | | | | | | | | | | |
| --- | --- | --- | --- | --- | --- | --- | --- | --- | --- | --- | --- | --- |
| Sonographer | Previous hysterectomy (%) | Rank | Left oophorectomy with intact uterus (%) | Rank | Tubal ligation (%) | Rank | Mean age (years) | Rank | Age at LMP (years) | Rank | Infertility (%) | Rank |
| 0 | 18.04 | 37 | 0.60 | 38 | 21.23 | 39 | 60.90 | 42 | 48.97 | 41 | 3.36 | 49 |
| 1 | 19.46 | 67 | 0.70 | 56 | 24.11 | 55 | 61.51 | 56 | 49.13 | 59 | 4.00 | 71 |
| 2 | 19.38 | 66 | 1.30 | 78 | 28.10 | 74 | 60.39 | 27 | 48.77 | 17 | 4.59 | 77 |
| 3 | 20.85 | 74 | 1.23 | 77 | 26.53 | 67 | 60.75 | 38 | 48.74 | 14 | 3.45 | 53 |
| 4 | 20.20 | 72 | 1.07 | 75 | 25.01 | 59 | 60.83 | 40 | 48.85 | 23 | 3.32 | 48 |
| 5 | 16.67 | 11 | 0.59 | 35 | 16.90 | 3 | 60.14 | 14 | 49.12 | 57 | 4.58 | 76 |
| 6 | 19.02 | 57 | 1.10 | 76 | 26.09 | 63 | 60.47 | 28 | 48.92 | 32 | 3.59 | 56 |
| 7 | 18.86 | 54 | 0.67 | 52 | 27.43 | 72 | 60.38 | 25 | 49.02 | 48 | 3.37 | 50 |
| 8 | 21.64 | 75 | 0.38 | 6 | 28.96 | 77 | 60.34 | 23 | 48.44 | 5 | 2.97 | 30 |
| 9 | 21.96 | 77 | 0.57 | 33 | 27.75 | 73 | 60.13 | 12 | 48.14 | 1 | 2.72 | 16 |
| 10 | 21.98 | 79 | 0.50 | 18 | 29.10 | 78 | 60.36 | 24 | 48.34 | 4 | 2.73 | 18 |
| 11 | 21.97 | 78 | 0.57 | 32 | 29.59 | 79 | 60.31 | 20 | 48.28 | 2 | 2.86 | 26 |
| 12 | 16.76 | 13 | 0.39 | 8 | 20.05 | 24 | 60.23 | 17 | 48.96 | 37 | 3.80 | 69 |
| 13 | 17.77 | 32 | 0.60 | 36 | 19.61 | 18 | 60.13 | 13 | 48.80 | 18 | 2.95 | 28 |
| 14 | 18.64 | 50 | 0.19 | 3 | 20.55 | 29 | 60.53 | 30 | 49.02 | 47 | 2.68 | 13 |
| 15 | 16.69 | 12 | 0.19 | 1 | 18.41 | 7 | 59.90 | 4 | 49.00 | 45 | 2.32 | 8 |
| 16 | 16.97 | 17 | 0.53 | 25.5 | 18.61 | 9 | 59.92 | 5 | 49.17 | 61 | 3.20 | 41 |
| 17 | 18.41 | 43 | 0.58 | 34 | 21.77 | 44 | 60.16 | 15 | 48.58 | 8 | 2.58 | 10 |
| 18 | 16.67 | 10 | 0.94 | 71 | 26.17 | 64 | 61.19 | 48 | 49.10 | 56 | 2.07 | 4 |
| 19 | 19.77 | 71 | 0.70 | 55 | 26.82 | 68 | 61.94 | 66 | 49.05 | 51 | 1.78 | 1 |
| 20 | 18.46 | 46 | 0.80 | 66 | 25.00 | 58 | 62.39 | 74 | 48.92 | 31 | 1.96 | 3 |
| 21 | 19.50 | 69 | 0.71 | 58 | 25.80 | 61 | 62.29 | 72 | 48.82 | 20 | 1.88 | 2 |
| 22 | 18.24 | 40 | 0.66 | 50 | 20.54 | 28 | 61.81 | 62 | 49.00 | 44 | 2.83 | 25 |
| 23 | 20.24 | 73 | 0.51 | 22 | 22.02 | 45 | 62.16 | 69 | 49.00 | 43 | 2.65 | 12 |
| 24 | 17.72 | 29 | 0.46 | 14 | 21.69 | 43 | 60.12 | 9 | 48.93 | 33 | 2.71 | 15 |
| 25 | 16.43 | 8 | 0.51 | 20 | 18.78 | 10 | 60.65 | 36 | 48.77 | 16 | 2.75 | 19 |
| 26 | 18.48 | 47 | 0.53 | 27 | 21.39 | 42 | 61.61 | 59 | 49.10 | 55 | 3.62 | 59 |
| 27 | 18.26 | 41 | 0.19 | 2 | 21.30 | 41 | 61.89 | 65 | 49.12 | 58 | 3.75 | 68 |
| 28 | 18.26 | 42 | 0.46 | 12 | 21.16 | 36 | 61.40 | 53 | 49.28 | 67 | 3.70 | 64 |
| 29 | 19.27 | 63 | 0.66 | 51 | 19.81 | 20 | 61.54 | 58 | 49.24 | 65 | 4.25 | 74 |
| 30 | 18.93 | 56 | 0.48 | 16 | 20.86 | 33 | 61.97 | 67 | 48.87 | 25 | 2.98 | 31 |
| 31 | 19.36 | 65 | 0.46 | 13 | 15.59 | 1 | 60.12 | 10 | 48.69 | 12 | 4.26 | 75 |
| 32 | 18.09 | 38 | 0.63 | 45 | 21.20 | 37 | 62.03 | 68 | 48.96 | 38 | 2.96 | 29 |
| 33 | 19.11 | 61 | 1.06 | 74 | 26.50 | 66 | 60.58 | 32 | 48.77 | 15 | 3.67 | 63 |
| 34 | 15.93 | 5 | 0.80 | 64 | 27.25 | 70 | 61.03 | 46 | 49.01 | 46 | 2.18 | 6 |
| 35 | 17.17 | 22 | 0.60 | 37 | 26.96 | 69 | 61.21 | 49 | 48.64 | 9 | 2.83 | 24 |
| 36 | 16.97 | 16 | 0.50 | 17 | 16.22 | 2 | 59.93 | 6 | 48.96 | 39 | 4.77 | 78 |
| 37 | 17.60 | 26 | 0.89 | 69 | 22.54 | 48 | 61.68 | 60 | 49.28 | 68 | 2.72 | 17 |
| 38 | 17.87 | 36 | 0.86 | 68 | 20.52 | 27 | 59.86 | 3 | 48.53 | 6 | 3.18 | 40 |
| 39 | 18.68 | 51 | 0.50 | 19 | 23.64 | 54 | 60.05 | 8 | 48.64 | 10 | 3.89 | 70 |
| 40 | 19.48 | 68 | 0.56 | 30 | 20.82 | 31 | 61.24 | 50 | 48.88 | 27 | 3.62 | 58 |
| 41 | 19.10 | 60 | 0.63 | 44 | 18.46 | 8 | 60.97 | 44 | 48.94 | 35 | 2.99 | 33 |
| 42 | 17.39 | 24 | 0.51 | 21 | 22.25 | 46 | 61.16 | 47 | 49.32 | 70 | 3.26 | 45 |
| 43 | 17.78 | 33 | 0.64 | 46 | 17.72 | 5 | 60.32 | 21 | 48.83 | 22 | 3.57 | 55 |
| 44 | 17.84 | 34 | 0.22 | 4 | 20.25 | 25 | 59.99 | 7 | 48.68 | 11 | 2.70 | 14 |
| 45 | 17.70 | 28 | 0.53 | 25.5 | 21.21 | 38 | 59.48 | 1 | 48.95 | 36 | 4.04 | 72 |
| 46 | 18.85 | 53 | 0.84 | 67 | 20.85 | 32 | 62.19 | 70 | 48.91 | 30 | 3.28 | 46 |
| 47 | 17.76 | 30 | 1.06 | 73 | 23.47 | 53 | 61.45 | 54 | 49.34 | 71 | 3.64 | 60 |
| 48 | 17.67 | 27 | 0.80 | 65 | 27.42 | 71 | 60.95 | 43 | 48.85 | 24 | 3.02 | 35 |
| 49 | 16.59 | 9 | 1.03 | 72 | 25.96 | 62 | 60.66 | 37 | 49.31 | 69 | 3.22 | 43 |
| 50 | 18.88 | 55 | 0.73 | 59 | 22.79 | 50 | 62.96 | 78 | 49.00 | 42 | 2.29 | 7 |
| 51 | 18.44 | 45 | 0.64 | 47 | 24.48 | 56 | 60.13 | 11 | 49.24 | 64 | 3.38 | 51 |
| 52 | 19.55 | 70 | 0.71 | 57 | 20.78 | 30 | 62.29 | 71 | 48.87 | 26 | 3.03 | 36 |
| 53 | 17.76 | 31 | 0.75 | 60 | 19.83 | 21 | 62.33 | 73 | 49.36 | 73 | 3.31 | 47 |
| 54 | 15.79 | 3 | 0.63 | 43 | 26.49 | 65 | 60.79 | 39 | 48.94 | 34 | 3.05 | 37 |
| 55 | 19.08 | 59 | 0.57 | 31 | 19.93 | 22 | 61.74 | 61 | 49.14 | 60 | 3.02 | 34 |
| 56 | 18.43 | 44 | 0.54 | 28 | 21.28 | 40 | 61.02 | 45 | 49.03 | 49 | 3.74 | 67 |
| 57 | 15.86 | 4 | 0.60 | 39 | 19.46 | 15 | 60.59 | 33 | 48.71 | 13 | 2.78 | 22 |
| 58 | 16.11 | 6 | 0.41 | 10 | 22.88 | 51 | 61.37 | 52 | 49.45 | 76 | 3.73 | 66 |
| 59 | 19.31 | 64 | 0.76 | 62 | 19.60 | 17 | 62.64 | 75 | 49.06 | 53 | 2.10 | 5 |
| 60 | 21.88 | 76 | 0.63 | 41 | 28.39 | 76 | 60.62 | 34 | 48.31 | 3 | 2.81 | 23 |
| 61 | 17.03 | 18 | 0.63 | 42 | 17.97 | 6 | 60.85 | 41 | 48.90 | 28 | 2.88 | 27 |
| 62 | 19.04 | 58 | 0.38 | 7 | 22.60 | 49 | 60.22 | 16 | 48.82 | 21 | 3.65 | 61 |
| 63 | 18.11 | 39 | 0.45 | 11 | 19.46 | 14 | 62.96 | 77 | 49.21 | 63 | 2.61 | 11 |
| 64 | 17.14 | 19 | 0.61 | 40 | 19.49 | 16 | 60.39 | 26 | 48.91 | 29 | 3.21 | 42 |
| 65 | 14.23 | 1 | 0.56 | 29 | 17.07 | 4 | 60.27 | 19 | 49.49 | 78 | 5.40 | 79 |
| 66 | 16.13 | 7 | 0.33 | 5 | 19.24 | 13 | 59.85 | 2 | 49.07 | 54 | 2.98 | 32 |
| 67 | 18.81 | 52 | 0.90 | 70 | 19.78 | 19 | 61.50 | 55 | 49.06 | 52 | 3.10 | 38 |
| 68 | 16.81 | 14 | 0.68 | 53 | 18.80 | 11 | 60.54 | 31 | 48.80 | 19 | 2.76 | 20 |
| 69 | 19.14 | 62 | 0.40 | 9 | 19.22 | 12 | 61.51 | 57 | 49.19 | 62 | 3.60 | 57 |
| 70 | 18.51 | 49 | 0.53 | 24 | 19.97 | 23 | 63.30 | 79 | 49.44 | 75 | 2.58 | 9 |
| 71 | 17.84 | 35 | 0.78 | 63 | 22.25 | 47 | 62.83 | 76 | 49.39 | 74 | 3.24 | 44 |
| 72 | 15.63 | 2 | 0.48 | 15 | 20.88 | 34 | 61.87 | 64 | 49.77 | 79 | 3.72 | 65 |
| 73 | 17.17 | 21 | 0.76 | 61 | 24.74 | 57 | 60.63 | 35 | 48.97 | 40 | 3.43 | 52 |
| 74 | 17.14 | 20 | 0.52 | 23 | 20.26 | 26 | 60.26 | 18 | 48.57 | 7 | 2.77 | 21 |
| 75 | 16.82 | 15 | 0.65 | 49 | 21.04 | 35 | 61.82 | 63 | 49.27 | 66 | 3.66 | 62 |
| 76 | 17.56 | 25 | 0.65 | 48 | 28.19 | 75 | 60.48 | 29 | 49.35 | 72 | 4.25 | 73 |
| 77 | 18.49 | 48 | 1.33 | 79 | 25.35 | 60 | 60.33 | 22 | 49.03 | 50 | 3.18 | 39 |
| 78 | 17.32 | 23 | 0.69 | 54 | 23.45 | 52 | 61.30 | 51 | 49.48 | 77 | 3.50 | 54 |

LMP, last menstrual period.
