# Supplementary material for: Quality assurance and its impact on ovarian visualization rates in the multicenter United Kingdom Collaborative Trial of Ovarian Cancer Screening (UKCTOCS)
Source: Ultrasound Obstet Gynecol. 2016 Feb 2;47(2):228–35. doi: 10.1002/uog.14929 (PMC4755159; doi:10.1002/uog.14929)
Supplement: Supplementary file 3 — Table S3 Mean baseline characteristics of 48 230 postmenopausal women scanned at one of 13 regional centers during the United Kingdom Collaborative Trial of Ovarian Cancer Screening [file UOG-47-228-s003.docx]

| Center | Previous hysterectomy (%) | Rank | Left oophorectomy with intact uterus (%) | Rank | Sterilization (%) | Rank | Infertility (%) | Rank | Mean age  (years) | Rank | Age at LMP (years) | Rank |
| --- | --- | --- | --- | --- | --- | --- | --- | --- | --- | --- | --- | --- |
| A | 16.63 | 3 | 0.62 | 7 | 19.03 | 3 | 2.71 | 2 | 60.53 | 6 | 48.72 | 4 |
| B | 19.22 | 12 | 0.45 | 1 | 15.84 | 1 | 4.45 | 12 | 59.96 | 1 | 48.66 | 2 |
| C | 18.67 | 9 | 0.52 | 5 | 22.42 | 9 | 3.00 | 6 | 60.13 | 3 | 48.72 | 3 |
| D | 18.78 | 11 | 1.03 | 13 | 26.48 | 12 | 3.57 | 9 | 60.56 | 7 | 49.01 | 9 |
| E | 18.41 | 6 | 0.64 | 9 | 20.72 | 6 | 3.63 | 10 | 60.85 | 8 | 48.97 | 8 |
| F | 18.03 | 5 | 0.51 | 4 | 21.76 | 8 | 3.74 | 11 | 61.68 | 10 | 49.29 | 11 |
| G | 13.60 | 1 | 0.68 | 11 | 17.25 | 2 | 5.12 | 13 | 60.32 | 4 | 49.56 | 13 |
| H | 18.64 | 8 | 0.64 | 10 | 20.80 | 7 | 3.04 | 7 | 62.08 | 11 | 49.03 | 10 |
| I | 18.54 | 7 | 0.53 | 6 | 20.23 | 5 | 2.86 | 3 | 62.28 | 13 | 49.30 | 12 |
| J | 21.99 | 13 | 0.49 | 3 | 28.98 | 13 | 2.88 | 4 | 60.35 | 5 | 48.28 | 1 |
| K | 17.25 | 4 | 0.47 | 2 | 19.06 | 4 | 3.12 | 8 | 60.10 | 2 | 48.93 | 6 |
| L | 18.74 | 10 | 0.75 | 12 | 25.01 | 10 | 1.92 | 1 | 62.14 | 12 | 48.96 | 7 |
| M | 16.24 | 2 | 0.62 | 8 | 26.21 | 11 | 2.88 | 5 | 60.88 | 9 | 48.91 | 5 |

**Table S3** Mean baseline characteristics of 48 230 postmenopausal women scanned at one of 13 regional centers during the United Kingdom Collaborative Trial of Ovarian Cancer Screening
