# Supplementary material for: Quality assurance and its impact on ovarian visualization rates in the multicenter United Kingdom Collaborative Trial of Ovarian Cancer Screening (UKCTOCS)
Source: Ultrasound Obstet Gynecol. 2016 Feb 2;47(2):228–35. doi: 10.1002/uog.14929 (PMC4755159; doi:10.1002/uog.14929)
Supplement: Supplementary file 4 — Table S4 Generalized estimating equation model for binary outcomes for comparison of observed and adjusted visualization rates (VR) of the right ovary on ultrasound at 13 regional centers participating in United Kingdom Collaborative Trial of Ovarian Cancer Screening, including non‐subjective factors identified previously as impacting on VR [file UOG-47-228-s004.docx]

**Table S4** Generalized estimating equation model for binary outcomes for comparison of observed and adjusted visualization rates (VR) of the right ovary on ultrasound (US) at 13 regional centers participating in United Kingdom Collaborative Trial of Ovarian Cancer Screening, including non-subjective factors identified previously as impacting on VR

| Variable | | OR (95% CI) | SE (robust) | *P* | Observed VR (%) | Adjusted VR  (% (95% CI))* | Rank based on observed VR | Rank based on adjusted VR |
| --- | --- | --- | --- | --- | --- | --- | --- | --- |
| Age at first US | | 0.961 (0.959–0.963) | 0.001 | 0.000 |  |  |  |  |
| Year of US | | 1.104 (1.099–1.108) | 0.002 | 0.000 |  |  |  |  |
| Age at LMP | | 1.008 (1.006–1.011) | 0.001 | 0.000 |  |  |  |  |
| Hysterectomy | | 0.472 (0.454–0.490) | 0.009 | 0.000 |  |  |  |  |
| Oopherectomy with intact uterus | | 0.199 (0.171–0.231) | 0.015 | 0.000 |  |  |  |  |
| Sterilization | | 0.898 (0.870–0.926) | 0.014 | 0.000 |  |  |  |  |
| BMI (normal group as reference) | |  |  |  |  |  |  |  |
| Underweight | 0.832 (0.725–0.954) | 0.058 | 0.009 |  |  |  |  |  |
| Overweight | 0.882 (0.857–0.908) | 0.013 | 0.000 |  |  |  |  |  |
| Obese | 0.696 (0.672–0.721) | 0.012 | 0.000 |  |  |  |  |  |
| Center | |  |  |  |  |  |  |  |
| A |  |  |  | 78 | 79 (78–80) | 4 | 4 |  |
| B | 1.141 (1.073–1.214) | 0.036 | 0.000 | 81 | 81 (80–82) | 3 | 3 |  |
| C | 0.489 (0.457–0.523) | 0.017 | 0.000 | 64 | 65 (64–66) | 11 | 11 |  |
| D | 0.786 (0.737–0.838) | 0.026 | 0.000 | 73 | 75 (74–76) | 7 | 7 |  |
| E | 0.476 (0.449–0.506) | 0.014 | 0.000 | 64 | 64 (63–65) | 12 | 12 |  |
| F | 1.488 (1.388–1.595) | 0.053 | 0.000 | 83 | 85 (84–86) | 1 | 1 |  |
| G | 0.904 (0.846–0.966) | 0.031 | 0.003 | 78 | 77 (76–78) | 5 | 5 |  |
| H | 0.571 (0.538–0.605) | 0.017 | 0.000 | 67 | 68 (67–69) | 8 | 8 |  |
| I | 0.427 (0.404–0.452) | 0.012 | 0.000 | 61 | 62 (61–63) | 13 | 13 |  |
| J | 0.547 (0.513–0.584) | 0.018 | 0.000 | 67 | 67 (66–68) | 9 | 9 |  |
| K | 1.169 (1.096–1.247) | 0.038 | 0.000 | 81 | 81 (81–82) | 2 | 2 |  |
| L | 0.543 (0.509–0.579) | 0.018 | 0.000 | 65 | 67 (66–68) | 10 | 10 |  |
| M | 0.863 (0.797–0.935) | 0.035 | 0.000 | 76 | 76 (75–78) | 6 | 6 |  |

*At mean covariate values. BMI, body mass index; LMP, last menstrual period; OR, odds ratio; SE, standard error.
